# Easy‐to‐Lay Poly‐N Heterocyclic Additives Enable Long‐Term Stabilization of Zinc‐Ion Capacitor Anodes under Deep Plating/Stripping

**DOI:** 10.1002/advs.202404323

**Published:** 2024-06-25

**Authors:** Yongfeng Bu, Qin Kang, Zhaomin Zhu, Hongyu Zhang, Yuman Li, Shihao Wang, Shengda Tang, Li Pan, Lijun Yang, Hongyu Liang

**Affiliations:** ^1^ Institute for Energy Research Jiangsu University Zhenjiang 212013 China; ^2^ Institute of Advanced Manufacturing and Modern Equipment Technology, School of Mechanical Engineering Jiangsu University Zhenjiang 212013 China; ^3^ Key Laboratory of Mesoscopic Chemistry of MOE, School of Chemistry and Chemical Engineering Nanjing University Nanjing 210023 China

**Keywords:** deep plating/stripping, dendrite‐free Zn anodes, electrolyte additives, high current density, Zn‐ion capacitors

## Abstract

Addition of organic compounds containing O/N heteroatoms to aqueous electrolytes such as ZnSO_4_ (ZS) solutions is one of the effective strategies to inhibit Zn anode dendrites and side reactions. However, addressing the stability of Zn plating/stripping at high current densities and areal capacities by this method is still a challenge, especially in capacitors known for high power and long life. Herein, an organic heterocyclic compound of 1, 4, 7, 10‐tetraazacyclododecane (TC) containing four symmetrically distributed N atoms is employed as ZS additive, expanding the life of Zn anodes from ≈ 30 h to 1000 and 240 h at deep plating/stripping conditions of 10 and 20 mA cm^−2^/mAh cm^−2^, respectively; the cumulative capacity is as high as 5.0 Ah cm^−2^ with 99% Coulombic efficiency, far exceeding reported additives. TC with higher binding energies than H_2_O for Zn species tends to adsorb to Zn (002) in a lying manner and participate in the solvation shell of Zn^2+^, thus avoiding Zn dendrites and side‐reaction damage, especially at high current densities. The TC‐endowed Zn anode's stability under such extreme conditions is verified in Zn‐ion capacitors (i.e., > 94.6% capacity retention after 28 000 cycles), providing new insights into the development of high‐power Zn‐based energy storage devices.

## Graphical abstract

An organic heterocyclic compound containing four symmetrically distributed N atoms has been proposed, which can substantially enhance the cycle life (up to 240–1000 h) of Zn anodes under deep plating/stripping conditions (10‐20 mA cm^−2^ and 10–20 mAh cm^−2^), along with high cumulative plated capacities of 2.4–5.0 Ah cm^−2^ and high Coulombic efficiency.

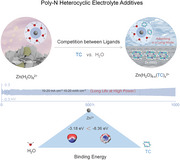

## 1 Introduction

With the increasing desire for high energy density and high‐safety energy storage devices, aqueous Zn‐batteries/capacitors (ZIBs/ZICs) have received unprecedented attention due to the high theoretical capacity (820 mAh g^−1^) of Zn anodes and the non‐flammability of aqueous electrolytes.^[^ ^1^ ^]^ The Zn and the electrolyte confer the above merits but substantially compromise the stability of the Zn anode system in operation, becoming a major challenge and hotspot for aqueous ZIBs/ZICs,^[^ ^2^, ^3^ ^]^ especially on ZICs that require higher operating current densities and longer lifetimes than ZIBs.^[^ ^4^ ^]^ The instability is mainly associated with the uncontrollability of crystal orientation during the Zn plating/stripping process (i.e., dendrite problems) and its high reactivity (i.e., side reactions) with the interfacial H_2_O from the electrolyte such as ZnSO_4_ (ZS).^[^ ^5^ ^]^ Typical strategies to address these concerns are mainly fromthe perspective of component and interface regulation of the Zn anode system involved, including the design of Zn anode components/morphology/structure,^[^ ^6^, ^7^, ^8^ ^]^ optimization of the Zn/ZS interface,^[^ ^9^, ^10^, ^11^, ^12^ ^]^separator modification,^[^ ^13^, ^14^ ^]^ and addition of electrolyte additives.^[^ ^15^, ^16^, ^17^, ^18^ ^]^Actually, the problems are more prominent for ZICs due to the additional high current density‐induced inhomogeneous electric field distribution.^[^ ^19^, ^20^ ^]^ The development of electrolyte additives is highly expected due to its very high potential for scale‐up applications and good compatibility with existing battery manufacturing,^[^ ^21^, ^22^ ^]^ especially due to its capability for both stabilizing the Zn/ZS interface and suppressing side reactions at high current densities.^[^ ^23^, ^24^ ^]^

Various additives for aqueous electrolytes have been developed explosively, typically including inorganic/organic salts,^[^ ^25^, ^26^ ^]^ organic small molecules/polymers,^[^ ^27^, ^28^ ^]^ and highly dispersible oxides.^[^ ^29^ ^]^ Ions dissociated from inorganic/organic salt additives modulate the Zn anode surface structure to promote Zn^2+^ to transfer through the electrostatic shielding effect of cations or competitive adsorption of anions, and thus they theoretically have the capability to enhance the operating current due to their compensation for carrier density,^[^ ^30^, ^31^ ^]^ but the actual current density/areal capacity of the devices involved is still at a low level (e.g., <2 mA cm^−2^/mAh cm^−2^ for MgSO_4_),^[^ ^32^ ^]^ as well as those using oxide additives (e.g., <5 mA cm^−2^/mAh cm^−2^ for SeO_2_).^[^ ^33^ ^]^ Organic macromolecules have the ability to capture the “host‐guest” structure through their unique cavities and long chains to obtain favorable crystalline surfaces. However, due to their large spatial potential resistance, the working current density is not ideal (e.g., <5 mA cm^−2^/mAh cm^−2^ for β‐cyclodextrin).^[^ ^34^ ^]^ Organic small molecule additives are mainly chain/cyclic structures with O/N only (e.g., tripropylene glycol)^[^ ^35^ ^]^ or O and N coexisting (e.g., glycine);^[^ ^36^ ^]^ some of these additives containing polyheteroatoms allow the devices to exhibit higher current density/areal capacity and cycle life (e.g., >10 mA cm^−2^/mAh cm^−2^ and >120 h for hexamethylenetetramine).^[^ ^37^ ^]^ The organic small molecule additives improve the stability of the Zn anode system mainly through the coordination of their electronegative O/N with Zn species;^[^ ^38^ ^]^ their specific adsorption on the Zn anode surface and their participation in the solvated structure of Zn^2+^ inhibit the growth of Zn dendrites and interfacial H_2_O activity, respectively, and thus stabilize the dynamic plating/stripping of Zn anodes.^[^ ^39^ ^]^ Undoubtedly, the molecular structure of organic additives, as well as the number and distribution of the O/N heteroatoms therein, determines their coordination with Zn species and the performance of the corresponding Zn anode systems and devices.

In this study, an organic heterocyclic compound of 1, 4, 7, 10‐tetraazacyclododecane (TC) containing four symmetrically distributed N atoms is proposed as a ZS additive, which confers Zn//Zn cells an extremely long cycle life of up to 1000 and 240 h at the ultrahigh current density and areal capacity of 10 and 20 mA cm^−2^ and mAh cm^−2^, respectively. The cumulative plated capacity of up to 5.0^ ^Ah cm^−2^ obtained under such deep plating/stripping conditions with >99% Coulombic efficiency (CE) is also the best performance reported so far for O/N‐containing organic additives. The electrostatic potential and binding energy of TC to Zn^2+^ are more negative than the competing ligand H_2_O due to its four symmetrically distributed N‐atoms with high affinity for Zn, which can preferentially adsorb to Zn (002) through the four N atoms in a lying mode while participate in the solvation layer of Zn^2+^, inhibit the 2 D diffusion of Zn^2+^, and further promote the exposure of the crystal surface of Zn (002), thereby inhibiting the persistent growth of Zn dendrites and the side reactions with interfacial H_2_O, especially at higher current densities. Such a stable Zn anode endowed by TC under harsh conditions is also verified in ZICs, i.e., 94.6% capacity retention after 28 000 cycles at high current densities, which is significantly better than the performance of other additives.

## 2 Results and Discussion

### 2.1 Adjustment of Zn^2+^ Solvation Structure

**Figure 1:**
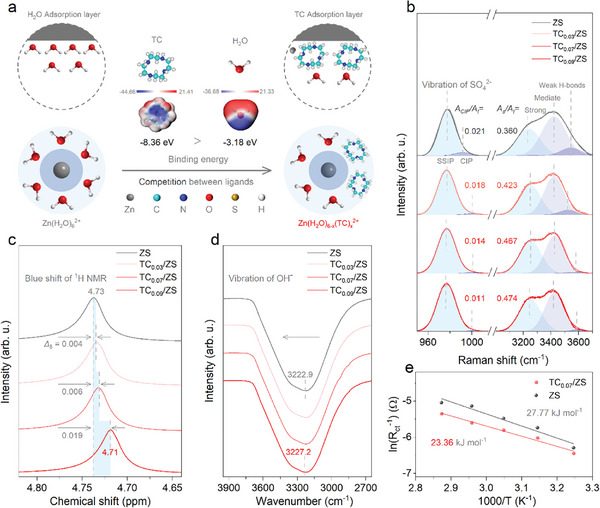
Effect of TC on the solvated structure of Zn^2+^ in TC/ZS. a) Schematic diagram of TC as a ligand competing with H_2_O in the solvated shell of Zn^2+^, i.e., the transition from Zn (H_2_O)_6_^2+^ to Zn (H_2_O)_6‐x_(TC)_x_^2+^, where x refers to the number of TC; insets are the chemical structure and electrostatic potential mapping of TC with a higher binding energy (−8.36 eV) than H_2_O (−3.18 eV). b) Raman spectra focused on the signals of SO_4_^2−^ (980 cm^−1^) and H─bonds (3000‐3800 cm^−1^). c,d) The corresponding ^1^H NMR and FTIR spectra. e) Arrhenius plots of Zn//Zn cells with TC_0.07_/ZS. The data related to ZS are provided for comparison.

**Figure** **1** presents the effect of TC on the solvation structure of Zn^2+^ in TC/ZS electrolyte. ZS is well known to be formed by the solvation of Zn^2+^ (i.e., the formation of Zn (H_2_O)_6_^2+^ by coordination with 6 H_2_O); the O in H_2_O serves as the coordinating atom. TC contains four N atoms, which are more likely to coordinate with Zn^2+^ due to the higher electrostatic potential mapping and binding energy (−8.36 eV vs −3.18 eV obtained by theoretical calculation).^[^ ^37^ ^]^ TC can efficiently modulate the solvated shell layer of Zn^2+^ by competition with the proto‐ligand H_2_O (Figure 1a),^[^ ^40^ ^]^ accordingly, resulting in the conversion from Zn (H_2_O)_6_^2+^ to Zn(H_2_O)_6‐x_(TC)_x_^2+^, as well as the regulation of the adsorption layer on Zn surface. In details, the fitted area ratio of strong H‐bonds (∼ 3250 cm^−1^) to total H‐bonds (*A*_s_/*A*_t_) increases with TC concentrations (i.e., from 0.360 to 0.423, to 0.467, and to 0.474, corresponding to ZS, TC_0.03_/ZS, TC_0.07_/ZS, and TC_0.09_/ZS, respectively) in the Raman spectra (the right of Figure 1b and Figure S1a, Supporting Information); in contrast, the ratio of mediate H‐bonds (3430 cm^−1^) and weak H‐bonds (≈ 3570 cm^−1^) decreases with the concentration of TC (Figure S1b, Supporting Information);^[^ ^41^, ^42^, ^43^ ^]^ meanwhile, the ratio of the contact ion pairs (CIP) to total (*A*_CIP_/*A*_t_) follows the same trend (i.e., decreasing from 0.021 to 0.011 with TC concentrations) for SO_4_^2−^ at 980 cm^−1^ (the left of Figures 1b and S1c,d, Supporting Information).^[^ ^44^, ^45^ ^]^ These results demonstrate that TC led to the formation of more stable clusters (Zn(H_2_O)_6‐x_(TC)_x_^2+^) by exchanging with some of the H_2_O and/or SO_4_^2−^ in the original cluster and reduces the amount of free H_2_O.^[^ ^27^, ^36^, ^46^, ^47^ ^]^ The strong interaction between TC and Zn^2+^ is also confirmed by the gradual blue shift (i.e., from 4.73 ppm to 4.71 ppm) of the ^1^H NMR signal of H_2_O in ZS, TC_0.03_/ZS, TC_0.07_/ZS, and TC_0.09_/ZS (Figure 1c),^[^ ^39^, ^48^ ^]^ as well as the varied position of ─OH from 3222.9 cm^−1^ to 3227.2 cm^−1^ in FTIR spectra (Figure 1d).^[^ ^49^, ^50^ ^]^

As expected, the desolation potential energy of Zn^2+^ in a typical TC_0.07_/ZS decreased from 27.77 kJ mol^−1^ in ZS to 23.36 kJ mol^−1^ (Figure 1e; Figure S2, Supporting Information),^[^ ^51^ ^]^ which facilitates the transfer of Zn^2+^ in TC_0.07_/ZS as evidenced by an increase in the transfer number from 0.23 in ZS to 0.39 in TC_0.07_/ZS (Figure S3, Supporting Information). Hence, the electrolyte of TC/ZS containing poly‐N TC additives will effectively regulate Zn plating/stripping and suppress interfacial side reactions by modulating the Zn^2+^ solvation structure,^[^ ^52^ ^]^ as highlighted below.

### 2.2 Mechanism of Zn Dendrite‐Free Growth with TC/ZS

**Figure 2:**
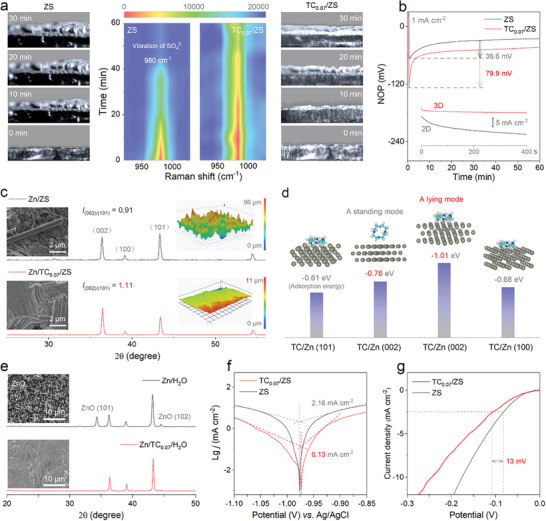
Mechanism of Zn dendrite‐free growth with TC/ZS. a) Cross‐section of an in situ optical image of Zn deposition (i.e., left and right panels) and the corresponding Raman spectra of the SO_4_^2−^ signal (i.e., centered panels). b) Initial nucleation overpotential of Zn^2+^ at 1 mA cm^−2^, 1 mAh cm^−2^; inset is the CA curve. c) XRD patterns of the Zn anode after 10 cycles at 10 mA cm^−2^, 10 mAh cm^−2^; insets are SEM images (left) and the corresponding UTM images (right). d) Adsorption energy of TC on Zn (002), (100), and (101) in a standing/lying mode. e) Further XRD and SEM of Zn soaked in TC_0.07_/H_2_O and H_2_O, respectively. f) Tafel curves. g) H_2_ evolution curves. For comparison, the data in ZS are also provided.

**Figure** **2** exhibits the evidence of highly reversible plating/stripping of Zn in TC/ZS. The Zn deposited in ZS at 5 mA cm^−2^ for only 10 min shows distinct protrusions observed by the cross‐section of in situ optical images, and the protrusions continued to increase with deposition time (the left of Figure 2a); in contrast, the one in TC_0.07_/ZS under the same conditions maintained a protrusion‐free and homogeneous surface throughout the deposition, including at 30 min (the right of Figure 2a). This significant depositional difference of Zn can be explained by the corresponding in situ Raman signal of counter‐ion SO_4_^2−^, i.e., TC_0.07_/ZS exhibits a more stable SO_4_^2−^ signal than ZS throughout the deposition, corresponding to the more uniform Zn^2+^ distribution on the anode interface (the middle of Figure 2a).^[^ ^53^ ^]^ The uniform and compact Zn deposition in TC_0.07_/ZS is supported by the higher initial nucleation overpotential (79.9 mV vs 36.6 mV, Figure 2b) than in ZS,^[^ ^54^, ^55^ ^]^ as well as the higher efficient diffusion mode (3 D vs 2 D, the inset of Figure 2b).^[^ ^56^, ^57^ ^]^ Hence, the TC with poly‐N heteroatoms in TC_0.07_/ZS effectively improves the kinetics of interfacial deposition reaction of Zn^2+^ and avoids the formation of dendritic protrusions due to concentration polarization.^[^ ^58^ ^]^

The XRD data of the Zn electrodes in Zn//Zn cell after cycling under deep plating/stripping conditions (i.e., 10 mA cm^−2^, 10 mAh cm^−2^) all exhibit typical Zn (002), (100), (101). However, the intensity ratio (*I*_(002)/(101)_ = 1.11) of Zn (002) to Zn (101) in TC_0.07_/ZS is significantly higher than that (1.11 vs 0.91) in ZS after 25 cycles (Figure 2c), indicating the exposure of Zn (002) after reversible Zn plating/stripping;^[^ ^59^ ^]^ the ex situ XRD data of the Zn anode during 0–25 cycling tests also confirm the result, i.e., the *I*_(002)/(101)_ increases from 0.53 to 1.11 as the number of cycles increase from 0 to 25 (Figure S4, Supporting Information). The resulting highly ordered surfaces with very small height fluctuations were observed by SEM and the corresponding UTM (the insets of Figures 2c and S5, Supporting Information). After 20 cycles, the Zn//Zn cell with ZS as the electrolyte was short‐circuited due to severe dendrites (Figure S6a,b, Supporting Information), whereas the one with TC_0.07_/ZS remained in a homogeneous and dense Zn (002) texture (Figure S6c,d, Supporting Information). Theoretical calculations confirm that TC tends to adsorb more on Zn surfaces than H_2_O, especially on Zn (002) due to higher adsorption energy (−0.76 eV vs −0.21 eV, Figures 2d and S7a, Supporting Information). It is worth noting that TC tends to adsorb on Zn (002) in a lying down mode rather than standing up due to its very negative adsorption energy of −1.01 eV; the corresponding charge transfer between N in TC and Zn is verified by the charge density difference (Figure S7b, Supporting Information). On this Zn surfaces with adsorbed TC, Zn^2+^ also has the smallest adsorption energy on Zn (002) (i.e., the exposed crystal planes with the lowest surface energy) compared to those on Zn (101) and Zn (100) (Figure S7c, Supporting Information), while its adsorption energy on the bare Zn surface fails to exhibit such a difference (Figure S7d, Supporting Information); the exposed Zn (002) has the lowest growth rate based on the Bravais law.^[^ ^28^, ^34^ ^]^ It is well agreement with the above SEM and XRD characterization. Experimental and theoretical results demonstrate that TC tends to adsorb on Zn (002) and induces Zn^2+^ to be able to grow stably along Zn (002) at a low speed due to the lowest surface energy, rather than disappearing due to growth at a faster rate as on Zn (101) and Zn (100).

The strong adsorption of TC on Zn is revealed by the difference in FTIR spectra of Zn in comparison experiments (i.e., Zn foils soaked in TC_0.07_/H_2_O versus in H_2_O, Figure S8, Supporting Information). The good wettability conferred by TC adsorption is observed from the decreased contact angle from 82.41° to 68.06° (Figure S9a,b, Supporting Information), which favors the reduction of the free energy of Zn^2+^ in contact with the interface.^[^ ^35^ ^]^ Further N 1 s and C 1 s XPS fine spectra reveal a significant Zn─N peak at 401.2 eV on Zn in addition to the typical C─N and N─H signals of TC (Figure S10a,b, Supporting Information),^[^ ^60^ ^]^ confirming that TC in H_2_O can be spontaneously adsorbed on Zn surfaces. Such a chemical environment is identical to that produced on the surface of the Zn electrodes involved in a Zn//Zn cell with 10 cycles (5 mA cm^−2^, 5 mAh cm^−2^),^[^ ^37^, ^61^ ^]^ demonstrating that TC in TC_0.07_/ZS can protect its surface during dynamic Zn plating/stripping (Figure S10c,d, Supporting Information). The surface of Zn adsorbed with TC (i.e., soaked in TC_0.07_/H_2_O) is relatively smooth in SEM images, and its XRD still has only typical Zn diffraction peaks (Figure 2e), whereas the surface of bare Zn (i.e., soaked in H_2_O) is disordered due to the formation of ZnO by side reactions.^[^ ^28^ ^]^ The significantly reduced corrosion current (i.e., from 2.16 to 0.13 mA cm^−2^) and increased corrosion potential of Zn in TC_0.07_/ZS compared to the case in ZS are direct evidences that TC is indeed effective in inhibiting the corrosion side reactions (Figure 2f). In addition, the reducing activity of interfacial H_2_O (i.e., the side reaction of H_2_ evolution) in TC_0.07_/ZS is effectively suppressed by TC as evidenced by a decrease in the H_2_ evolution potential of ≈13 mV (Figure 2g). TC is an organic compound that can be stabilized in aqueous ZS solutions; no significant redox peaks were observed even for the devices involved in the voltage range of 0–1.8 V. (i) TC can modulate its solvation structure by coordinating with Zn^2+^; it not only reduces the desolvation energy of the proposed plating Zn^2+^, but also reduces the content of reactive H_2_O carried to the interface of the Zn anode; (ii) TC also competes with H_2_O for preferentially coordinating with the Zn^2+^ produced by stripping, which inhibits the side reactions in the plating and stripping of the Zn anode, thus enhancing the cycling stability of Zn anodes. Therefore, this TC additive containing poly‐N heteroatoms tends to preferentially adsorb on Zn (002) in a lying mode to efficiently inhibit dendrites and side reactions in the dynamic plating/stripping of Zn.

### 2.3 Superior Stability of Zn Anodes under Deep Plating/Stripping Conditions

**Figure 3:**
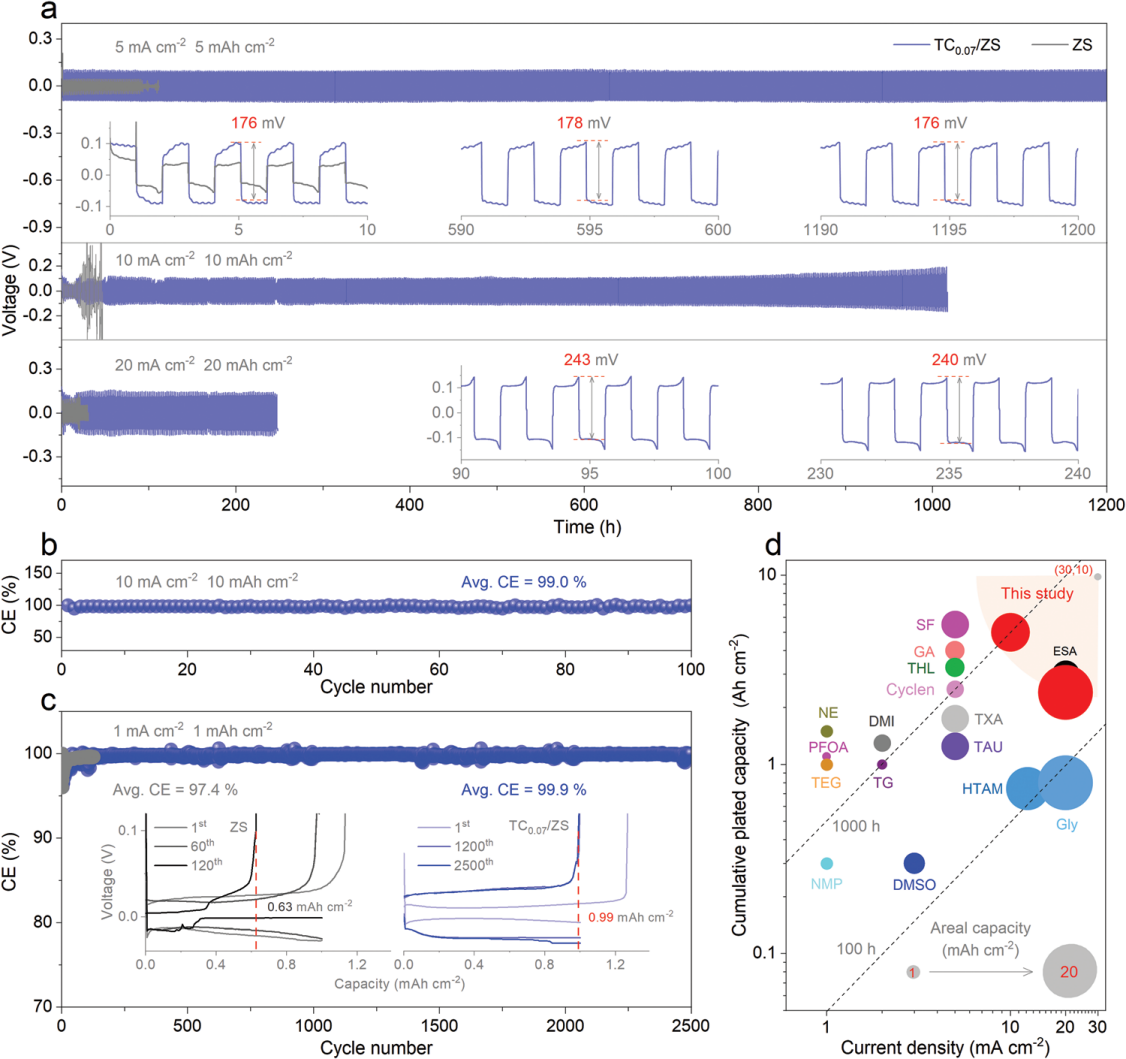
Superior long‐term stability of Zn//Zn cells and CE of Zn//Cu cells with TC_0.07_/ZS. a) Cycling stability at 5–20 mA cm^−2^, 5–20 mAh cm^−2^; insets are the extracted typical charge/discharge curve. b,c) CE performance under low (i.e., 1 mA cm^−2^, 1 mAh cm^−2^) and high (i.e., 10 mA cm^−2^, 10 mAh cm^−2^) charge/discharge conditions of Zn//Cu cells; insets of c) are typical charge/discharge curves. d) The state‐of‐the‐art performance in terms of current density and cumulative plated capacity compared to the reported similar additive systems. The data in ZS are also provided for comparison.

Based on the Zn//Zn cells at a relatively high test condition (5 mA cm^−2^, 5 mAh cm^−2^), TC_0.07_/ZS has the longest cycling life of up to > 1200 h compared to ZS (92 h), TC_0.03_/ZS (256 h), and TC_0.09_/ZS (674 h) (the top of Figures 3a and S11a, Supporting Information), demonstrating that the concentration of TC in TC_0.07_/ZS is optimal (i.e., 0.07 mol L^−1^); note that this concentration of TC has little effect on the ionic conductivity and viscosity of TC_0.07_/ZS (Figure S11b,c, Supporting Information). In fact, in a relatively low condition (e.g., 2 mA cm^−2^, 1 mAh cm^−2^), its cycling life is longer up to >2000 h (Figure S12a, Supporting Information), while in an extreme condition its life is relatively reduced. For instance, the cycling life is >1000 h at the deep plating/striping conditions of 10 mA cm^−2^, 10 mAh cm^−2^ (the middle of **Figure** **3**a), and >240 h at the deeper conditions of 20 mA cm^−2^, 20 mAh cm^−2^ (the bottom of Figure 3a); ZS as a control sample, barely works for 22–30 h under the same conditions. The corresponding cumulative plated capacities under such harsh conditions are as high as 5.0 and 2.4 Ah cm^−2^, which are the best performance of any additive known to date. Although the polarization voltage of the charge/discharge curve involved is higher than that of ZS due to the slightly increased viscosity (i.e., caused by the strong interaction between TC and Zn^2+^),^[^ ^62^ ^]^ it is very stable (176‐178 mV and 240–243 mV) throughout the whole cycling test at both low and high current densities (insets of Figure 3a). It is worth noting that the Zn//Zn cell operating at such high rates can still be easily converted to low‐rate conditions (e.g., from 10 to 0.5 mA cm^−2^) for stable operation (Figure S12b, Supporting Information). In addition, Zn//Zn cells with TC_0.07_/ZS still exhibits a long cycling life (> 800 h) after 5 days of placement at room temperature; in contrast, those with ZS at the same placement duration only cycle for 14 h (Figure S12c, Supporting Information), indicating that the TC additive is effective in inhibiting Zn corrosion and side‐reactions, even during transportation/storage of the device. Such high stability (i.e., >240/1000 h) conferred by TC under such deep plating/striping conditions (10/20 mA cm^−2^, 10/20 mAh cm^−2^) is far superior to some of the typical N‐only (e.g., HMTA)^[^ ^37^ ^]^ or N/O‐containing (e.g., Gly, ESA)^[^ ^36^, ^63^ ^]^ additives claiming high power capability reported previously.

In terms of the effect of TC_0.07_/ZS on the device CE, the Zn//Cu cell was employed. Under typical extreme plating/stripping conditions (10 mA cm^−2^, 10 mAh cm^−2^), the cells using TC_0.07_/ZS not only cycled stably for a long period of time (> 100 cycles, corresponding to ∼ 200 h) but also had a very high avg. CE (99.0%) (Figure 3b). On the contrary, the cells using ZS could hardly operate under such conditions, let alone cycle stably. As expected, under a relaxed cycling condition (1 mA cm^−2^, 1 mAh cm^−2^), TC_0.07_/ZS endows the cell with a higher avg. CE (99.9%) and cycle life (>2500 cycles, corresponding to ∼5000 h), which are also significantly higher than that (97.4%, 120 cycles) of ZS (Figure 3c). In the corresponding voltage profile, the curves of 1^st^‐2500^th^ are not only smooth, where the one of 1200^th^ also almost coincides with the 2500^th^, corresponding to a reversible Zn stripping capacity of 0.99 mAh cm^−2^ at the Cu electrode due to the inhibition of dendrite growth and side reactions, whereas the curve of 120^th^ starts to lose their smoothness characteristics in the case of the cells using ZS, with a corresponding capacity of only 0.63 mAh cm^−2^ (the insets of Figures 3c and S13, Supporting Information). Anyway, only in terms of current density or cumulative plated capacity the TC in this study can be compared to the reported top levels of N/O‐containing Gly,^[^ ^64^ ^]^ ESA,^[^ ^63^ ^]^ SF,^[^ ^52^ ^]^ and GA,^[^ ^65^ ^]^ etc. (Figure 3d). However, intaking both into account, TC imparts to Zn//Zn cells a comprehensive performance that significantly outperforms that of the typical organic additives that have been reported so far (Table S1, Supporting Information), i.e., much closer to the desirable orange region centered at 30 mA cm^−2^, 10 Ah cm^−2^.

### 2.4 Verification of TC_0.07_/ZS in ZICs

**Figure 4:**
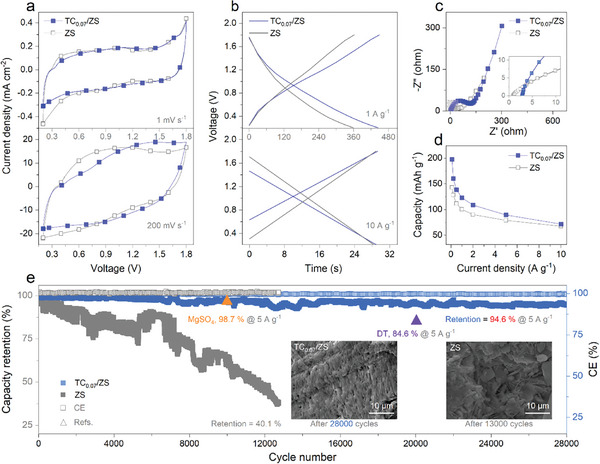
Capacitance performance of Zn^2+^ capacitors with TC_0.07_/ZS as electrolyte. a) CV curves at 1 mV s^−1^ (the upper half) and 200 mV s^−1^ (the lower half), respectively. b) The corresponding GCD curves at 1 A g^−1^ and 10 A g^−1^. c) Nyquist plots; the inset is an enlarged view focused on the gray area. d) Evolution of capacity with current density. e) Cycling stability of devices up to 28 000 cycles at 5 A g^−1^; insets are the corresponding SEM images after cycling test. All data with ZS as the electrolyte are also provided for comparison.

ZICs with activated carbon as cathode were constructed (Figure S14a,b, Supporting Information) to evaluate the effect of TC_0.07_/ZS on their capacitance, rate and cycling stability, as shown in **Figure** **4**. At 1 mV s^−1^, the CV curve of TC_0.07_/ZS almost coincides with that of ZS except that the cathodic H_2_ evolution current is significantly reduced, both of which are of approximate rectangular shapes (the top of Figure 4a), indicating that the addition of TC does not alter the original electric double‐layer capacitance behavior.^[^ ^66^, ^67^ ^]^ Moreover, the voltage window of the ZICs involved is not compressed (i.e., 0.2–1.8 V); in fact, the cathodic voltage window can be expanded a bit due to the suppression of H_2_O reduction side reactions by TC.^[^ ^20^ ^]^ With the increase from 1 mV s^−1^ to the higher 200 mV s^−1^, the CV curves of ZICs with either TC_0.07_/ZS or ZS as the electrolyte gradually deviate from the original shape into a shuttle shape between 0.2 and 1.8 V (the bottom of Figure 4a; Figure S15a,b, Supporting Information).^[^ ^68^ ^]^ However, the deviation of TC_0.07_/ZS is relatively large, i.e., the rate capacity decays more due to the slightly smaller area enclosed by the corresponding CV curves.^[^ ^69^ ^]^ As expected, the voltage window of the GCD curves for TC_0.07_/ZS at 1 A g^−1^ is consistent with that of the CV curves, but the charging/discharging time is indeed longer than that of ZS (i.e., higher capacity at small currents). At a high current density of 10 A g^−1^, the charging/discharging time shrinks to a comparable level (Figure 4b; Figure S15c, Supporting Information), and the voltage drop of TC_0.07_/ZS in the GCD curves is slightly larger due to the adsorption of TC on Zn anodes with respect to those of ZS, which is in line with the trend of the evolution of the CV curves at the different scanning rates described above. The detrimental effect caused by TC was also confirmed by the electrochemical impedance results (Figure 4c), i.e., increased solution resistance (from 0.97 to 3.1 Ω) and interfacial charge transfer resistance (from 50 to 122 Ω) with respect to ZS. The former is due to the increase in viscosity induced by the coordination of TC with Zn^2+^ (Figure S11c, Supporting Information),^[^ ^20^ ^]^ while the latter is associated with the strong adsorption of TC at the Zn anode interface.

ZICs with TC_0.07_/ZS as the electrolyte can release up to 197 mAh g^−1^ at the minimum current density of 0.1 A g^−1^, which is significantly higher than that (143 mAh g^−1^) of ZS under the same conditions (Figure 4d); even if the current density is increased by a factor of 100 to the ultra‐high 10 A g^−1^ (this is a very high current density for ZICs with carbon as the cathode), the capacity (72 vs 65 mAh g^−1^) is still slightly higher than that of ZS, i.e., the capacity is higher than ZS over the entire range of 0.1–10 A g^−1^.

For ZICs, in order to further improve their rate performance, we have improved them by increasing the pore size of activated carbon (Figure S16a, Supporting Information), decreasing the areal mass loading of carbon electrode (Figure S16b, Supporting Information), and decreasing the interfacial resistance between current collector and activated carbon by spraying Au (Figure S16c, Supporting Information), but none of them are effective in increasing the rate of ZICs. It demonstrates that the rate performance of ZICs is mainly limited by the plating/stripping kinetics of Zn anodes rather than carbon cathodes and the solid‐solid interfaces involved, highlighting the importance of developing aqueous electrolyte additives due to their great potential for Zn anode plating/stripping modulation. In the development of aqueous electrolyte additives, there is a need not only to improve cycling stability, but preferably also to increase the rate of Zn//Zn cells or ZICs or to sacrifice rate as little as possible.

Accordingly, despite the addition of TC results in increased resistance to ion and electron transport in the system, its suppression of interfacial side reactions still confers a higher rate capacity for ZICs (this is the most promising result in the study of high‐power devices like capacitors). In fact, 10 A g^−1^ is already the highest level of ZICs reported in various literatures (Table S2, Supporting Information); moreover, ZICs with TC_0.07_/ZS electrolyte in this study also have high capacity retention at the same current density. As expected, ZICs using TC_0.07_/ZS as electrolyte have a long cycle life of >28 000 charge/discharge cycles with a capacity retention as high as 94.6% at 5 A g^−1^ and 99.9% CE, whereas the capacity of ZS has decayed significantly after 13 000 cycles with a capacity retention of only 40.1% (Figure 4e). Such high capacity retention and number of cycles at high current densities conferred by the TC proposed in this study are indeed far superior to the reported typical additives for ZICs, such as 84.6% for D‐trehalose dihydrate (DT) at 5 A g^−1^ and only 10 000 cycles for MgSO_4_ at 5 A g^−1^, respectively.^[^ ^32^, ^65^ ^]^ In fact, even after being subjected to 28 000 cycles, the surface of the Zn anode with TC_0.07_/ZS exhibits relatively uniform Zn plating due to the homogeneous Zn^2+^ adsorption conferred by TC over the entire electrode surface (the inset of Figure 4e); on the contrary, the surface of the Zn anode with additive‐free ZS is full of disorganized dendritic accumulations after only 13 000 cycles.

## 3 Conclusion

In summary, a poly‐N heterocyclic compound TC suitable for ZS aqueous electrolyte additives was proposed and its optimal concentration was determined to be 0.07 mol L^−1^. The unique ring‐like structure of TC containing four symmetrically distributed N atoms is significantly different from reported additives containing only single/few N/O heteroatoms (e.g., N in ─NH_2_ and O in ─COOH/OH/C─O─O─C). What's more, its ability to dramatically enhance the stability of Zn anodes under deep plating/stripping conditions was fully verified. It has higher binding/adsorption energy for Zn species compared to the competing ligand, H_2_O; this allows TC to adsorb to Zn (002) preferentially in a lying mode while participating in the solvation shell of Zn^2+^, thus suppressing dendrite growth and side‐reactions under deep plating/stripping conditions. TC provides more Zn^2+^ plating sites and proveing its great ability to stabilize Zn anodes under deep plating/stripping conditions at high current densities. The TC_0.07_/ZS‐endowed dendrite‐free Zn anode system can tolerate extreme plating/stripping conditions up to 10–20 mA cm^−2^ and 10–20 mAh cm^−2^; its stable lifetime and cumulative plated capacity are even more 240–1000 h and 2.4–5.0 Ah cm^−2^, respectively, which outperforms other reported organic additives. Its capacity, rate, and lifetime performance in ZICs also validate the superiority of TC additives. This study demonstrates that the TC is suitable for Zn anode systems that require deep plating/stripping at high current densities, which sheds light on the design of ZS aqueous electrolytes required for high‐power ZICs.

## 4 Experimental Section

### Preparation and Characterization of Electrolytes

ZnSO_4_ of 2 mol L^−1^ aqueous solution was prepared by dissolving appropriate concentrations of ZnSO_4_·7H_2_O (Sinopharm, AR) in deionized H_2_O. The modified electrolytes were prepared by adding different concentrations of 1, 4, 7, 10‐tetraazacyclododecane (TC, Aladdin, 97%) into 2 mol L^−1^ ZnSO_4_. Subsequently, they were stirred separately for 6 h to obtain a homogeneous solution for use. Finally, electrolytes containing different concentrations of TC (i.e., 0.03, 0.07, and 0.09 mol L^−1^) were obtained and denoted as TC_0.03_/ZS, TC_0.07_/ZS, and TC_0.09_/ZS, respectively. The electrolyte was analyzed by Raman spectra (LabRAM Aramis, a laser with an excitation wavelength of 532 nm), Fourier transform infrared spectrometer (FTIR, Thermo Scientific Nicolet iS50), and Hydrogen nuclear magnetic resonance spectrometer (^1^H NMR, BRUKER AVANCE 400, with DMSO). The viscosity was measured with a rheometer (Anton Paar MCR 302, Austria).

### Electrochemical Measurements

Glass fiber separator absorbed 80 µL electrolyte (Whatman D) and two pre‐cut Zn foils (thickness: 200 µm, φ: 12 mm, 99.99%) were assembled to Zn//Zn cells using CR2032 packs. In the same procedure, Zn//Cu cells was assembled with glass fiber separators and Cu foils as the cathode (thickness: 30 µm, φ: 12 mm, 99.99%).

The cathodes in ZICs were prepared by mixing activated carbon with rich in micropores (Figure S14a) with acetylene black and polytetrafluoroethylene in a mass ratio of 8:1:1, then adding ethanol and milling thoroughly to form a stable slurry, which was then pressed onto a carbon fiber cloth collector with a diameter of 12 mm to form a flat‐surface sheet electrode (Figure S14b, Supporting Information) and dried at 60 °C. The mass loading of the cathode was 1.3‐1.4 mg cm^−2^. TC_0.07_/ZS and ZS were employed as electrolyte in ZICs. The Zn anodes were cut from pre‐prepared Zn foils. The involved activated carbon, Zn foils, and Cu foils were obtained from Cyber Electrochemical Materials Network.

Tafel plots were measured between −0.7 and −1.1 V at 1 mV s^−1^ with Zn foils and platinum wires as the working and counter electrodes, respectively. Ag/AgCl was employed as the reference electrode. The hydrogen evolution reaction (HER) tests for the ZS and TC_0.07_/ZS electrolytes were performed with two Zn foils in a two‐electrode configuration. The Chronoamperograms (CA) curves were measured at a fixed potential of −1.2 V and a current density of 5 mA cm^−2^. These tests were done on the electrochemical workstation (IVIUM, Vertex One‐C).

### Calculation Methods

The calculations were obtained from the framework of the density functional theory with the projector augmented plane‐wave method. The generalized gradient approximation proposed by Perdew et al. was selected for the exchange‐correlation potential. The long‐range van der Waals interaction was constructed by the DFT‐D3 approach. The cut‐off energy for plane wave was 450 eV; the energy criterion was 10^−5^ eV in iterative solution of the Kohn‐Sham equation. A vacuum layer of 15 Å was added perpendicular to the sheet to avoid artificial interaction between periodic images. The Brillouin zone integration was performed using a 3 × 3 × 1 k‐mesh for Zn (002) and (101) surface, and 3 × 2 × 1 k‐mesh for Zn (100) surface. The structures were relaxed until the residual forces on the atoms have declined to < 0.03 eV Å^−1^.

### Characterization Methods

In situ Raman tests were performed on a homemade transparent Raman electrolytic cell using a Zn foil symmetrical electrode. The signal was obtained by integrating over five scans during discharge at 5 mA cm^−2^. In order to focus the laser directly on the surface of the Zn working electrode, the distance between the working electrode and the quartz window was set less than 100 µm. The Raman spectrometer was operated using a laser power of 100 mW and a wavelength of 532 nm.

The micromorphology of Zn deposition was revealed via scanning electron microscope (SEM, JSM‐7800F). The crystal structure of the plated/stripped Zn were detected by X‐ray diffraction (XRD, Bruker D8, German) with Cu Kα in the scan range of 5–80° (2 θ) with a step size of 0.07°. The surface compositions of Zn anodes were investigated by X‐ray photoelectron spectroscopy (XPS, Thermo Scientific K‐Alpha). The roughness of Zn electrodes in different electrolytes was recorded by an ultra‐depth three‐dimensional microscope (UTM, KEYENCE VHX‐1000). The in situ observations of Zn plating/stripping were carried out on an optical microscope (60 X‐100 X) equipped with a magnifying glass holder. All characterizations were performed at room temperature of 25 °C unless otherwise stated.

## Conflict of Interest

The authors declare no conflict of interest.

## Supporting information

Supporting Information

## Data Availability

The data that support the findings of this study are available from the corresponding author upon reasonable request.
