# Supplementary material for: Easy‐to‐Lay Poly‐N Heterocyclic Additives Enable Long‐Term Stabilization of Zinc‐Ion Capacitor Anodes under Deep Plating/Stripping
Source: Adv Sci (Weinh). 2024 Jun 25;11(32):2404323. doi: 10.1002/advs.202404323 (PMC11348090; doi:10.1002/advs.202404323)
Supplement: Supplementary file 1 — Supporting Information [file ADVS-11-2404323-s001.docx]

**Supporting Information**

**Easy-to-Lay Poly-N Heterocyclic Additives Enable Long-Term Stabilization of Zinc-Ion Capacitor Anodes under Deep Plating/Stripping**

*Yongfeng Bu,^a#^* Qin Kang,^a#^ Zhaomin Zhu,^a^ Hongyu Zhang**,^a^ Yuman Li,^a^ Shihao Wang,^a^ Shengda Tang,^b^ Li Pan,^b^ Lijun Yang,^c^ Hongyu Liang^b^**

*^a^**Institute for Energy Research, Jiangsu University, Zhenjiang 212013, China*

*^b^**Institute of Advanced Manufacturing and Modern Equipment Technology, School of Mechanical Engineering, Jiangsu University, Zhenjiang 212013, China*

*^c^Key Laboratory of Mesoscopic Chemistry of MOE, School of Chemistry and Chemical Engineering, Nanjing University, Nanjing 210023, China*

*E-mail addresses:* yfbu@ujs.edu.cn; hyliang@ujs.edu.cn

^#^These authors contributed equally to this work.

**Contents:**

**Supplementary Figures and Tables: Figures S1-S16 and Tables S1-S2**

**Figure S1** Raman spectra and H-bonding interaction of different TC/ZS electrolytes.

**Figure S2** EIS of Zn//Zn cells at different temperatures.

**Figure S3** Measurement of Zn^2+^ transfer number.

**Figure S4** Ex-situ XRD data of the Zn anode after 0, 5, 10, 15, 20 and 25 cycles, respectively at 10 mA cm^-2^ and 10 mAh cm^-2^.

**Figure S5** XRD and UTM characterizations for pristine Zn foil.

**Figure S6** SEM of Zn electrodes in Zn//Zn cells after 22 cycles.

**Figure S7** DFT theoretical calculation for absorption energy.

**Figure S8** FTIR spectra of TC and Zn foils soaked in TC_0.07_/H_2_O and H_2_O for 10 days.

**Figure S9** Contact angles of ZS and TC_0.07_/ZS electrolyte on Zn foils.

**Figure S10** XPS fine spectra of Zn foils soaked in different electrolytes.

**Figure S11** Optimization of electrolyte concentration.

**Figure S12** Cyclic stability of Zn//Zn cells under different testing conditions.

**Figure S13** Voltage-capacity plots of Zn//Cu cells with TC_0.07_/ZS and ZS electrolytes.

**Figure S14** Structure characterization of the cathode.

**Figure S15** CV and GCD curves of ZICs with TC_0.07_/ZS and ZS as electrolytes.

**Figure S16** Results of attempted measures used to increase the rate of ZICs.

**Table S1** Superiority of TC over reported organic additives.

**Table S2** Comparison for the rate performance of ZICs using similar additives.


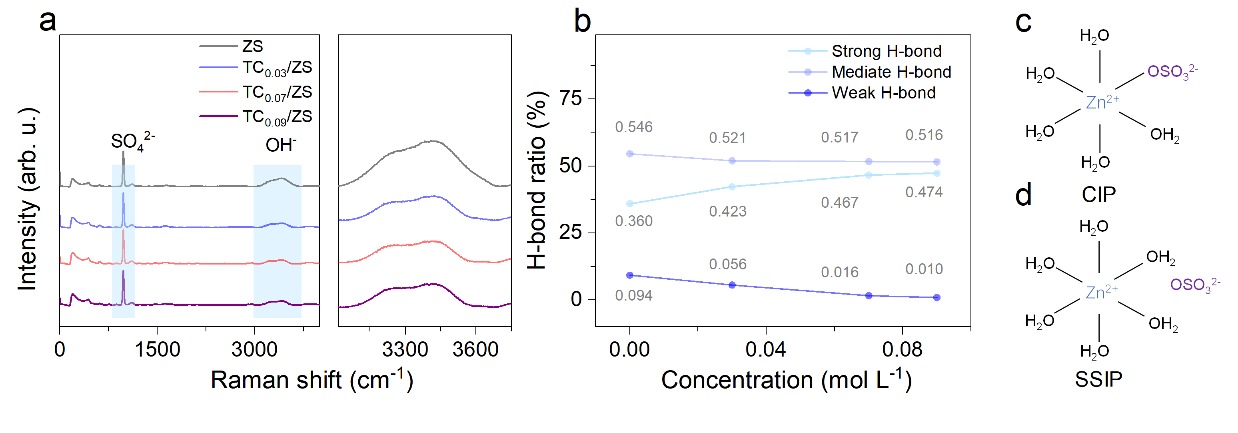


**Figure S1.** Raman spectra and H-bonds interaction of different TC/ZS electrolytes. a) Raman spectra of ZS, TC_0.03_/ZS, TC_0.07_/ZS, and TC_0.09_/ZS, respectively; b) the ratios of strong, medium, and weak H-bonds; c,d) Zn^2+^ structures of the contact ion pairs (CIP) and the solvent-separated ion pairs (SSIP), respectively.

The broad peak of the -OH at 3000-3700 cm^-1^ can be further deconvoluted into three components of strong (∼ 3250 cm^-1^), mediate (∼ 3430 cm^-1^), and weak H-bonds (∼ 3570 cm^-1^). The fitted area ratios of strong H-bonds to total H-bonds increase with TC concentration, while the ratios of medium and weak H-bonds to total H-bonds all decrease with TC concentrations.

Based on the classical Eigen-Tamm (ET) mechanism, the strong peak of SO_4_^2-^ can be divided into two pairs of contact ion pairs (CIP) and solvent-separated ion pairs (SSIP), respectively. Since TC replaces SO_4_^2-^ in the TC/ZS electrolyte, it is difficult to enter the solvation shell of Zn^2+^, so the CIP ratio (*A*_CIP_/*A*_t_) decreases with TC concentrations.


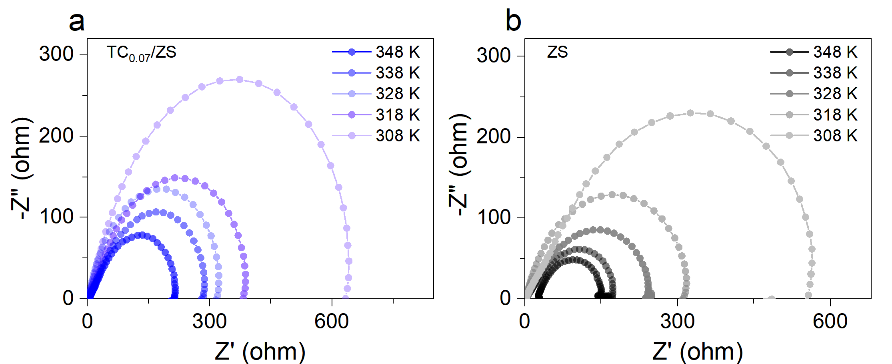


**Figure S2.** EIS of Zn//Zn cells at different temperatures. a) Using TC_0.07_/ZS as electrolyte; b) using ZS as electrolyte.

The EIS of Zn//Zn cells with a temperature range of 308-348 K was used to measure the desolation potential energy (*E*_a_) of the charge transfer process at the Zn surface. EIS was tested at the frequency range of 10^5^-10^-2^ Hz with the voltage amplitude of 5 mV. The distribution of relaxation time (DRT) analysis was performed by using DRT Tools.^[1]^

The *E*_a_ was calculated based on the Arrhenius equation:^[2]^

$$\frac{lnT}{R_{CT}}=\frac{E_{a}}{RT}+lnA$$

Where *R*_ct_ is the charge transfer resistance, *A* is the frequency factor, *R* is the gas constant, and *T* is the absolute temperature.


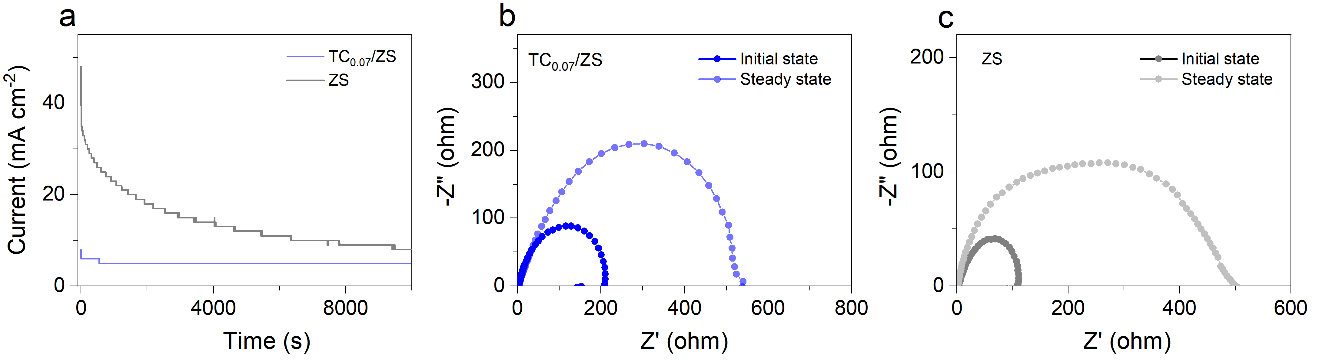


**Figure S3.** Measurement of Zn^2+^ transfer number. a) Current variation in Zn//Zn cells at a constant potential of 15 mV; b,c) EIS of initial state and steady state for TC_0.07_/ZS and ZS.

Measurement of Zn^2+^ transfer number uses Zn//Zn cells at a constant potential of 15 mV and EIS before polarization (initial state) and after 10000 s of polarization (steady state). The transfer number of Zn^2+^ (*t*) can be calculated by the following equation.^[3]^

$$t_{{Zn}^{2+}}=\frac{I_{s}(\Delta V-I_{0}R_{0})}{I_{0}(\Delta V-I_{s}R_{s})}$$

Where ∆𝑉 (15 mV) is the applied constant potential; 𝐼_0_ and 𝐼𝑠 are the initial and steady-state response current, respectively. 𝑅_0_ and 𝑅𝑠 are the electrode interface impedances before and after the polarization, respectively.


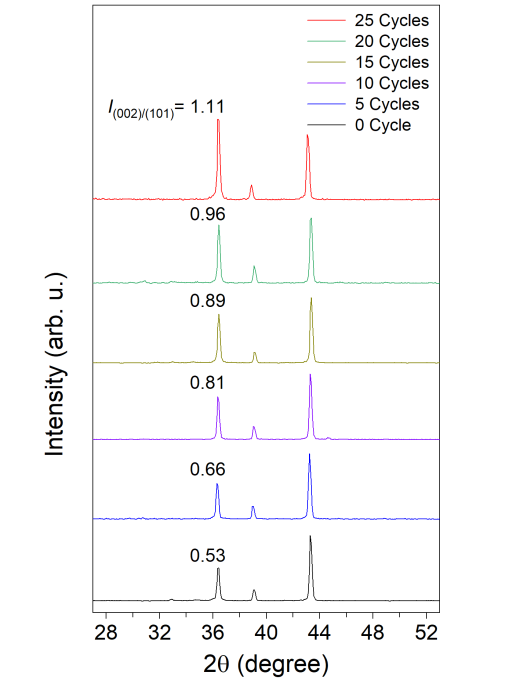


**Figure S4.** Ex-situ XRD data of the Zn anode after 0, 5, 10, 15, 20 and 25 cycles, respectively at 10 mA cm^-2^ and 10 mAh cm^-2^.


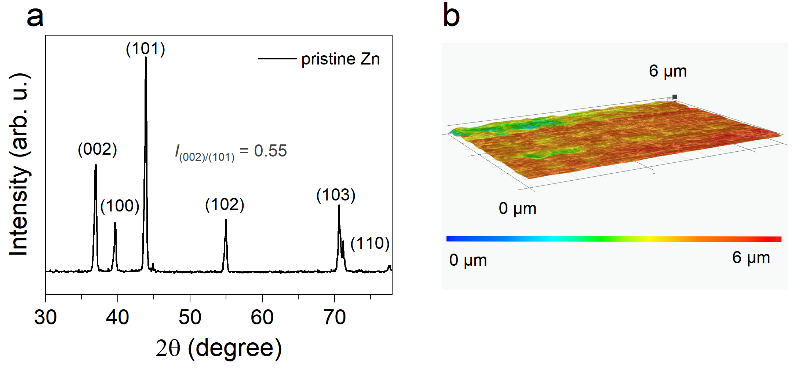


**Figure S5.** Characterization for pristine Zn foil. a) XRD; b) UTM.

The intensity ratio (i.e., *I*_(002)/(101)_ = 1.11) of Zn (002) to Zn (101) in TC_0.07_/ZS is significantly higher than that (0.55) of pristine Zn; its height fluctuates very slightly with respect to pristine Zn. But in ZS, the height fluctuation is very high.


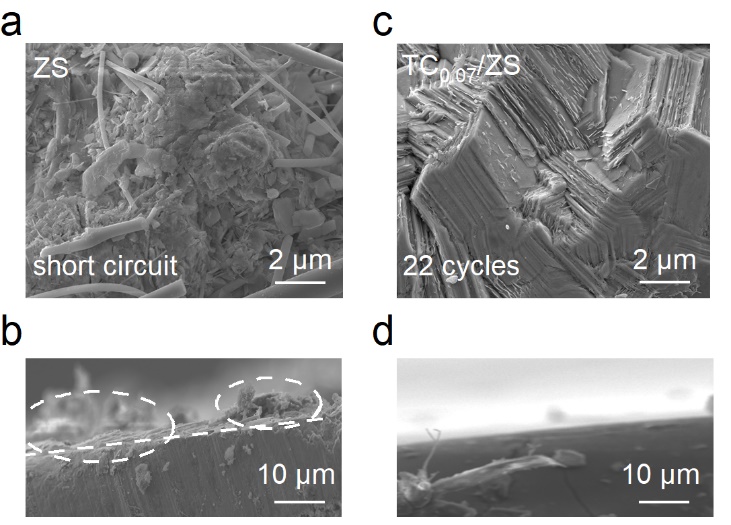


**Figure S6.** SEM of Zn electrodes in Zn//Zn cells after 22 cycles. a,b) With ZS as the electrolyte and the corresponding cross section, respectively; c,d) with TC_0.07_/ZS as electrolyte and the corresponding cross-section, respectively.


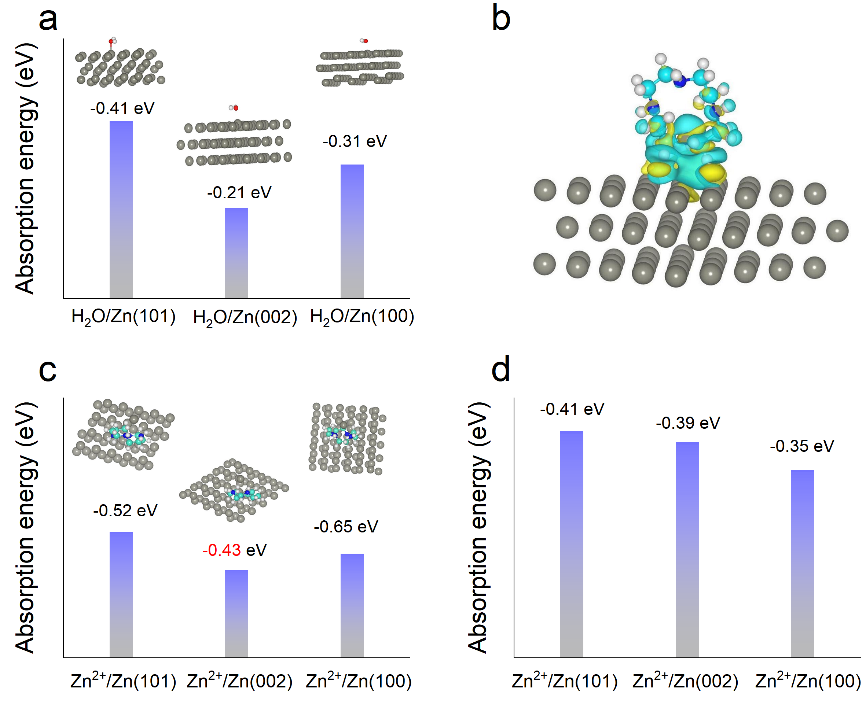


**Figure S7.** DFT theoretical calculation for absorption energy. a) H_2_O adsorbed on Zn (101), Zn (002), and Zn (100); b) charge density difference of TC adsorbed on the Zn (002); c) Zn atoms on different Zn crystal planes after adsorption of TC; d) Zn atoms on different Zn crystal planes.

Zn atoms have the smallest adsorption energy (i.e., lowest surface energy) for Zn (002) with TC adsorption. Based on the Bravais law, this implies that the slowest growth rate on Zn (002), thus exhibiting the crystalline features of Zn (002) after cycling. On the contrary, Zn (101) and Zn (100) disappeared due to faster growth rates.


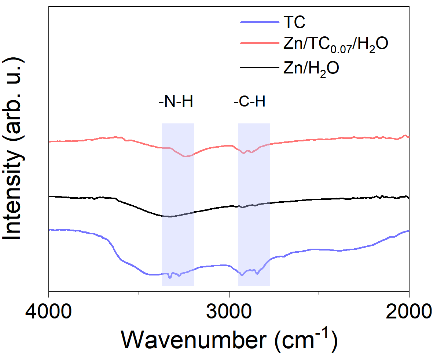


**Figure S8.** FTIR spectra of TC and Zn foils soaked in TC_0.07_/H_2_O and H_2_O for 10 days.

FTIR spectra of Zn foil soaked in TC_0.07_/H_2_O shows the N-H and C-H peaks of TC, indicating the adsorption of TC on the surface of Zn foils.

**
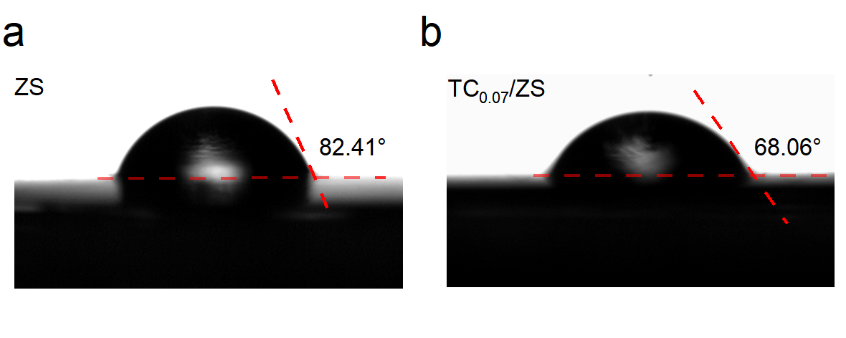
**

**Figure S9.** Contact angles of a) ZS electrolyte and b) TC_0.07_/ZS electrolyte on Zn foils.


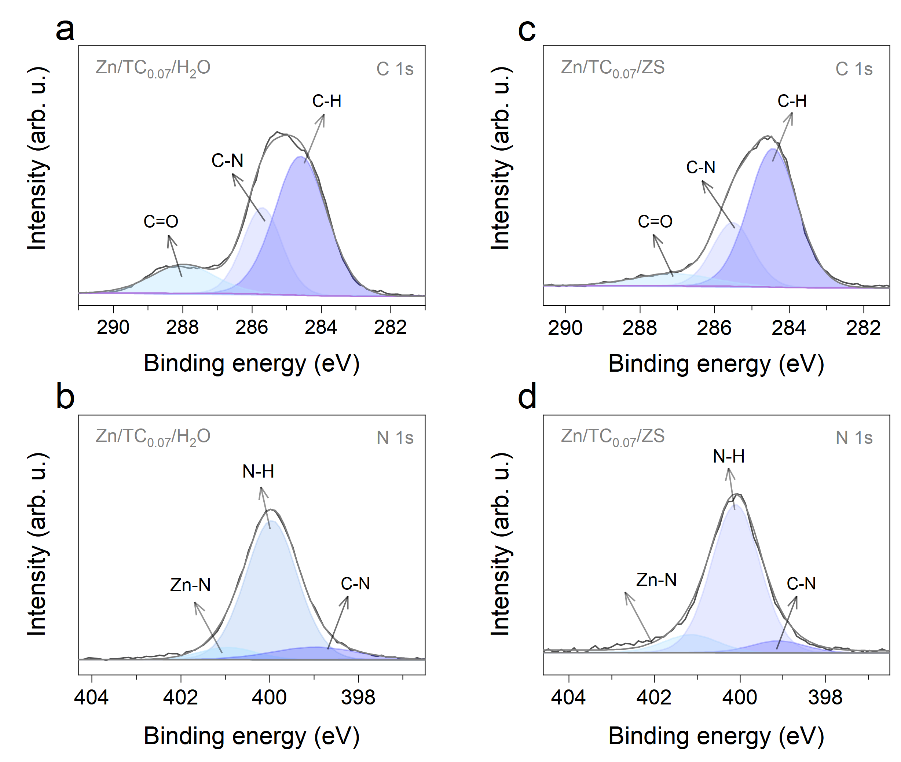


**Figure S10.** XPS fine spectra of Zn foils soaked in different electrolytes. a,b) C 1s and N 1s obtained in TC_0.07_/H_2_O; c,d) C 1s and N 1s obtained in TC_0.07_/ZS after 10 cycles at 5 mA cm^-2^ and 5 mAh cm^-2^.

The C 1s and N 1s spectra of Zn foils soaked in TC_0.07_/H_2_O for 10 days are very close to that of Zn foils used in Zn//Zn cells with TC_0.07_/ZS after 10 cycles. There is an important chemical bond of Zn-N, in addition to the presence of C-H, C-N C=O, N-H, and C-N peaks in C 1s and N 1s, indicating that the Zn foil surfaces under both conditions have very similar chemical environments.

**
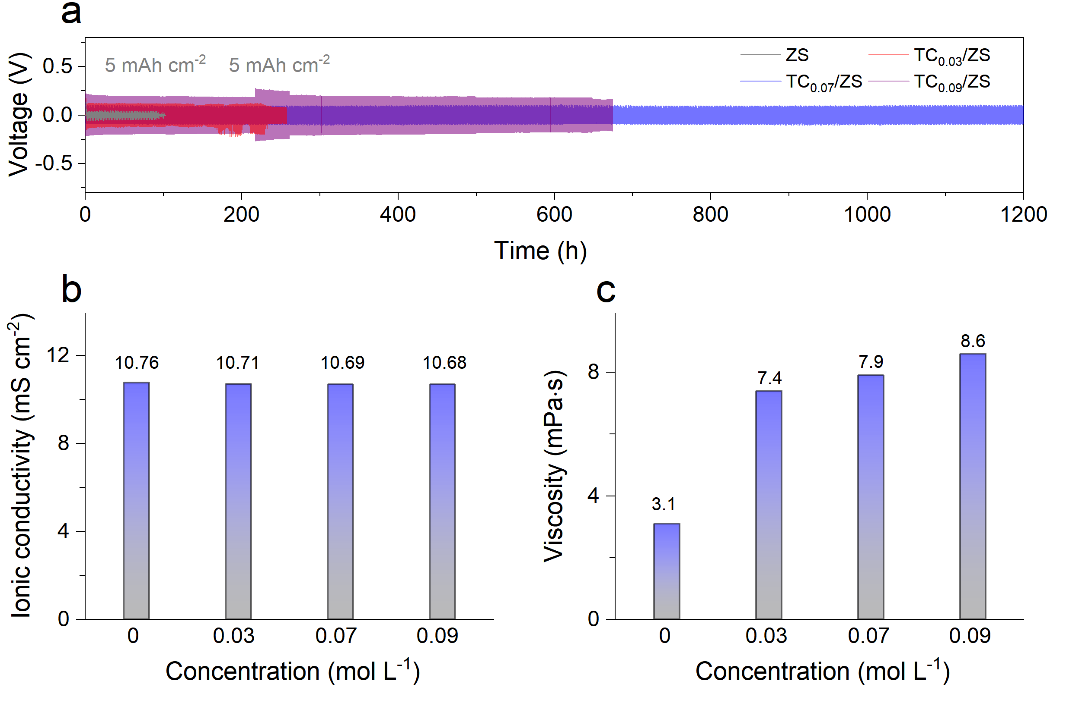
**

**Figure S11.** Optimization of electrolyte concentrations. a) Cycling stability of Zn//Zn cells with different concentrations of TC electrolytes at 5 mA cm^-2^ and 5 mAh cm^-2^; b,c) the corresponding ionic conductivity and viscosity, respectively.

**
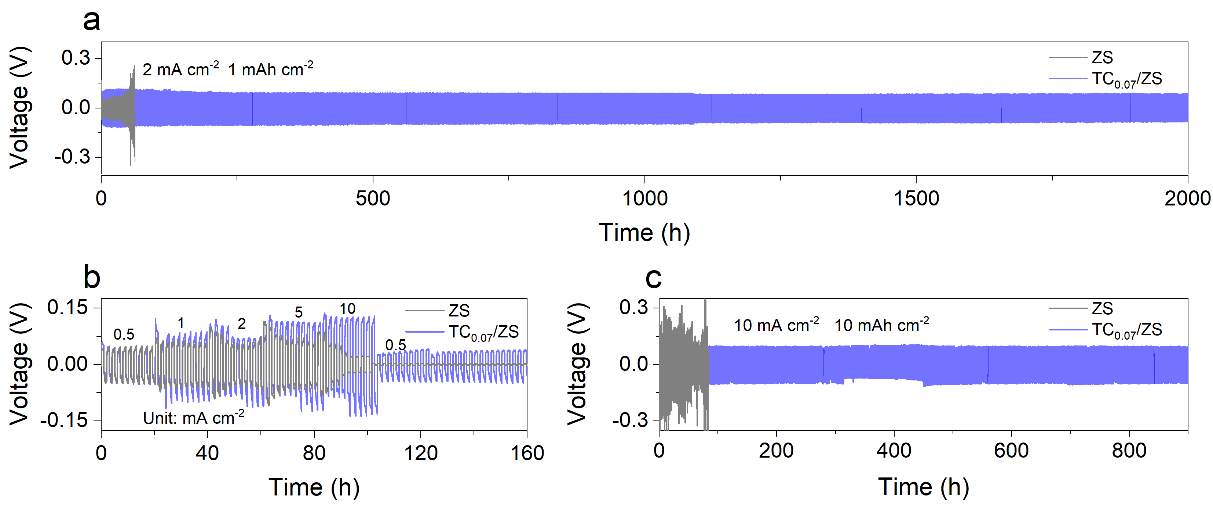
**

**Figure S12**. Cyclic stability of Zn//Zn cells under different testing conditions. a) At 2 mA cm^-2^, 1 mAh cm^-2^; b) the rate performance at different current densities and areal capacities; c) after 5 days of placement at room temperature with TC_0.07_/ZS electrolyte.


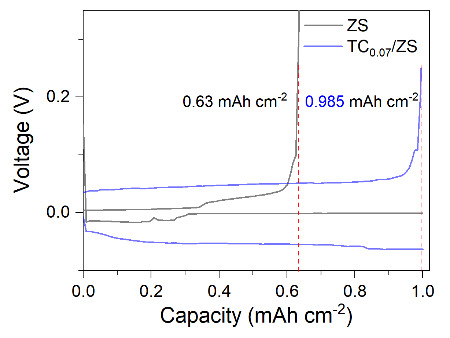


**Figure S13**. Voltage-capacity plots of Zn//Cu cells with TC_0.07_/ZS and ZS electrolytes.


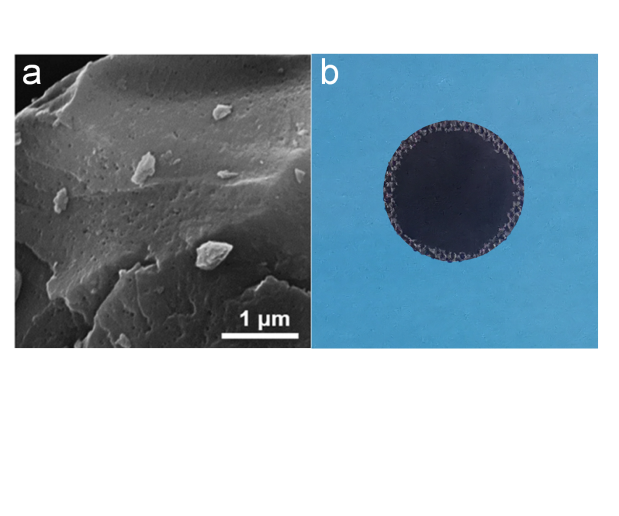


**Figure S14**. Structure characterization of the cathode. (a) A typical SEM image of the cathode; (b) morphology of the prepared cathode.


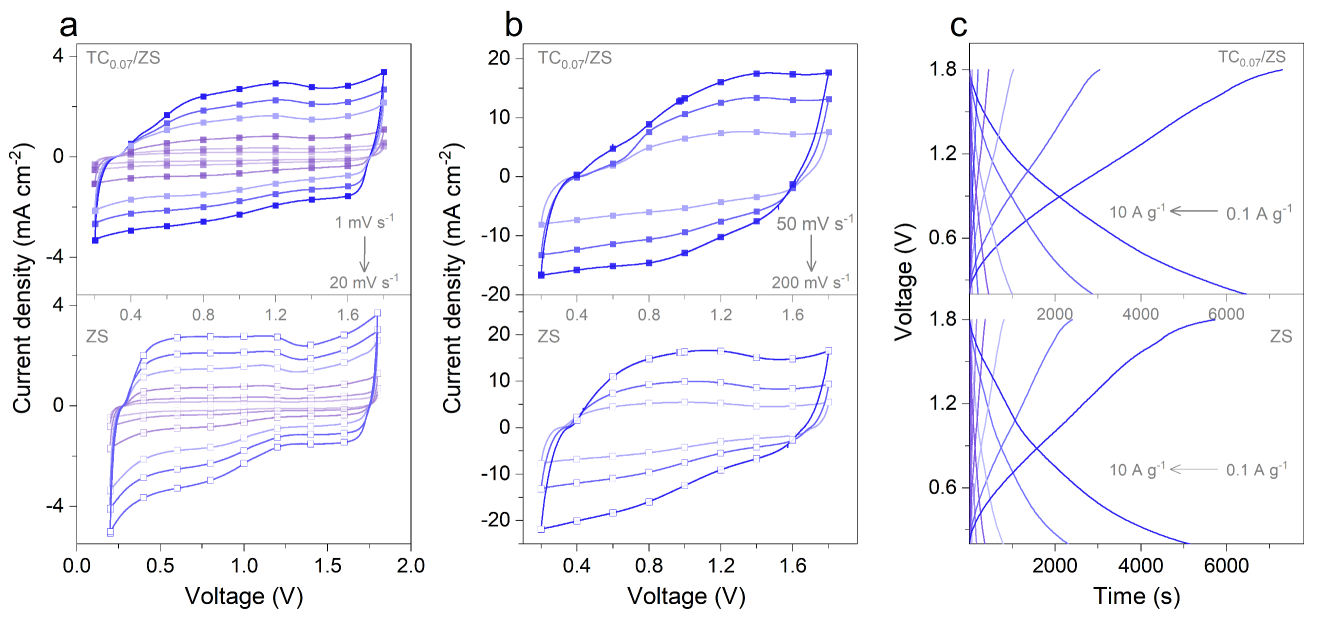


**Figure S15**. CV and GCD curves of ZICs with TC_0.07_/ZS and ZS electrolytes. a,b) CV curves at 1-20 mV s^-1^ and at 50-200 mV s^-1^, respectively; c) GCD curves at 0.1-10 A g^-1^.


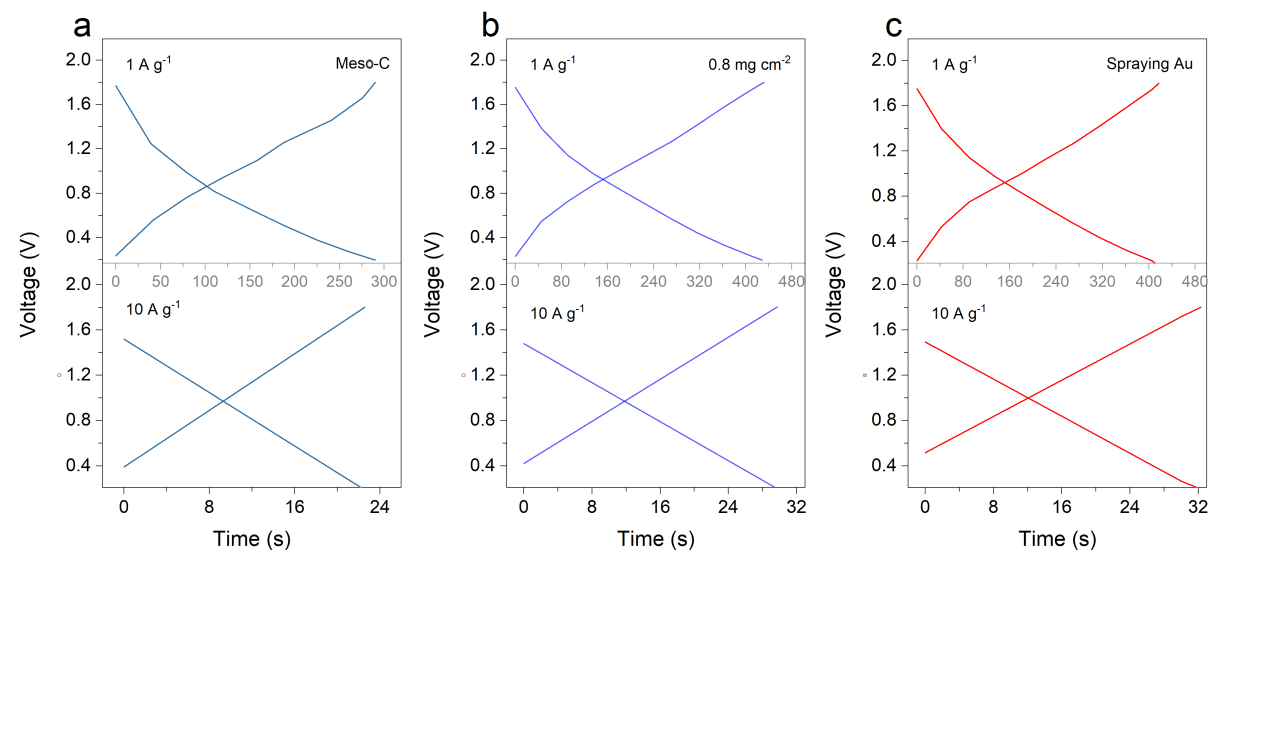


**Figure** **S16** Results of attempted measures used to increase the rate of ZICs. a) Using mesopore-dominated activated carbon as active material; b) decreasing the areal mass loading from 1.4 to 0.8 mg cm^-2^; c) spraying Au on current collectors.

**Table S1.** Superiority of TC over reported organic additives in terms of plating/stripping conditions, cycle life, and cumulative capacity.

| Electrolyte | Additives | Current density (mA cm^-2^) | Areal capacity  (mAh cm^-2^) | Life time (h) | Cumulative capacity  (Ah cm^-2^) | Refs. |
| --- | --- | --- | --- | --- | --- | --- |
| 2 mol L^-1^ ZnSO_4_ | TC | 10 | 10 | 1000 | 5 | This study |
|  |  | 20 | 20 | 240 | 2.4 |  |
|  | SF | 5 | 5 | 2200 | 5.5 | ^[4]^ |
|  | GA | 5 | 1 | 1600 | 4 | ^[5]^ |
|  | THL | 5 | 2.5 | 1300 | 3.25 | ^[6]^ |
|  | ESA | 20 | 5 | 300 | 3 | ^[7]^ |
|  | TXA | 5 | 5 | 700 | 1.75 | ^[8]^ |
|  | NMP | 1 | 1 | 600 | 0.3 | ^[9]^ |
|  | Gly | 20 | 20 | 80 | 0.8 | ^[10]^ |
|  | HMTA | 12.4 | 12.4 | 120 | 0.744 | ^[11]^ |
|  | DMI | 3 | 3 | 1300 | 1.3 | ^[12]^ |
|  | TG | 2 | 0.67 | 1000 | 1 | ^[13]^ |
|  | DMSO | 3 | 3 | 200 | 0.3 | ^[14]^ |
|  | NE | 1 | 1 | 3000 | 1.5 | ^[15]^ |
|  | PFOA | 1 | 0.5 | 2200 | 1.1 | ^[16]^ |
|  | TEG | 1 | 1 | 2000 | 1 | ^[17]^ |
| 2 mol L^-1^ Zn(otf)_2_ | TAU | 5 | 2.5 | 1600 | 4 | ^[18]^ |

**Table S2**. Comparison for the rate performance of ZICs using similar additives.

| Electrolyte | Additives | Capacity (mAh g^-1^) | | Cycles@ current density (A g^-1^) | retention (%) | Refs. |
| --- | --- | --- | --- | --- | --- | --- |
|  |  | @ 1 A g^-1^ | @ 10 A g^-1^ |  |  |  |
| 2 mol L^-1^ ZnSO_4_ | TC | 128 | 72 | 28000@ 5 | 94.6 | This study |
|  | M | 87 | 60 | 25000@ 5 | 90 | ^[19]^ |
|  | DT | 85 | 60 | 20000@ 5 | 84.6 | ^[20]^ |
|  | CMC | 88 | 61.1@ 5 | 30000@ 2 | 97 | ^[21]^ |
|  | MgSO_4_ | 154 | 100@ 5 | 10000@ 5 | 98.7 | ^[22]^ |

**References**

1. C. Li, R. Kingsbury, A. S. Thind, A. Shyamsunder, T. T. Fister, R. F. Klie, K. A. Persson, L. F. Nazar, *Nat. Commun.* **2023**, *14*, 3067.
2. H. Pan, Y. Shao, P. Yan, Y. Cheng, K. S. Han, Z. Nie, C. Wang, J. Yang, X. Li, P. Bhattacharya, K. T. Mueller, J. Liu, *Nat. Energy* **2016**, *1*, 16039.
3. C. Meng, W. He, L. Jiang, Y. Huang, J. Zhang, H. Liu, J. J. Wang, *Adv. Funct. Mater.* **2022**, *32*, 2207732.
4. L. Zhang, T. Zhang, W. Xin, H. Peng, Z. Yan, Z. Zhu, *Mater. Today Energy* **2022**, *29*, 101130.
5. H. Zheng, Y. Huang, J. Xiao, W. Zeng, X. Li, X. Li, M. Wang, Y. Lin, *Chem. Eng. J.* **2023**, *468*, 143834.
6. H. Li, Y. Ren, Y. Zhu, J. Tian, X. Sun, C. Sheng, P. He, S. Guo, H. Zhou, *Angew. Chem. Int. Ed.* **2023**, *62*, e202310143.
7. Y. Wang, R. Zhao, M. Liu, J. Yang, A. Zhang, J. Yue, C. Wu, Y. Bai, *Adv. Energy Mater.* **2023**, *13*, 2302707.
8. J. Yin, H. Liu, P. Li, X. Feng, M. Wang, C. Huang, M. Li, Y. Su, B. Xiao, Y. Cheng, X. Xu, *Energy Stor. Mater.* **2023**, *59*, 102800.
9. Y. Ma, Q. Zhang, L. Liu, Y. Li, H. Li, Z. Yan, J. Chen, *Nat. Sci. Rev.* **2022**, *9*, nwac051.
10. Y. Liu, Y. An, L. Wu, J. Sun, F. Xiong, H. Tang, S. Chen, Y. Guo, L. Zhang, Q. An, L. Mai, *ACS Nano* **2022**, *17*, 552-560.
11. H. Yu, D. Chen, Q. Li, C. Yan, Z. Jiang, L. Zhou, W. Wei, J. Ma, X. Ji, Y. Chen, L. Chen, *Adv. Energy Mater.* **2023**, *13*, 2300550.
12. K. Lu, C. Chen, Y. Wu, C. Liu, J. Song, H. Jing, P. Zhao, B. Liu, M. Xia, Q. Hao, W. Lei, *Chem. Eng. J.* **2023**, *457*, 141287.
13. Z. Liu, R. Wang, Q. Ma, J. Wan, S. Zhang, L. Zhang, H. Li, Q. Luo, J. Wu, T. Zhou, J. Mao, L. Zhang, C. Zhang, Z. Guo, *Adv. Funct. Mater.* **2023**, *34*, 2214538.
14. D. Feng, F. Cao, L. Hou, T. Li, Y. Jiao, P. Wu, *Small* **2021**, *17*, 2103195.
15. L. Zhang, L. Miao, W. Xin, H. Peng, Z. Yan, Z. Zhu, *Energy Stor. Mater.* **2022**, *44*, 408-415.
16. F. Zhao, Z. Jing, X. Guo, J. Li, H. Dong, Y. Tan, L. Liu, Y. Zhou, R. Owen, P. R. Shearing, D. J. L. Brett, G. He, I. P. Parkin, *Energy Stor. Mater.* **2022**, *53*, 638-645.
17. Y. Li, J. Cheng, D. Zhao, X. Chen, G. Sun, S. Qiao, W. Zhang, Q. Zhu, *Energy Stor. Mater.* **2023**, *63*, 102997.
18. J. Yang, Y. Zhang, Z. Li, X. Xu, X. Su, J. Lai, Y. Liu, K. Ding, L. Chen, Y. P. Cai, Q. Zheng, *Adv. Funct. Mater.* **2022**, *32*, 2209642.
19. X. Wang, H. Peng, K. Sun, F. Yang, Z. Liu, S. Cui, X. Xie, G. Ma, *Energy Stor. Mater* **2024**, *66*, 103208.
20. H. Peng, X. Wang, F. Yang, Z. Liu, H. Lei, S. Cui, X. Xie, G. Ma, *Chem. Eng. J.* **2023**, *474*, 145864.
21. H. Huang, J. Yun, H. Feng, T. Tian, J. Xu, D. Li, X. Xia, Z. Yang, W. Zhang, *Energy Stor. Mater* **2023**, *55*, 857-866.
22. P. Wang, X. Xie, Z. Xing, X. Chen, G. Fang, B. Lu, J. Zhou, S. Liang, H. J. Fan, *Adv. Energy Mater.* **2021**, *11*, 2101158.
